# Histiocytic Sarcoma Arising in a Background of Nodular Lymphocyte Predominant Hodgkin Lymphoma

**DOI:** 10.1155/crh/9949707

**Published:** 2025-07-23

**Authors:** Gul Emek Yuksek Wymer, Susana Ferra, Mohtashim Naeem

**Affiliations:** Department of Pathology and Laboratory Medicine, HCA Florida Healthcare, Plantation, Florida, USA

**Keywords:** histiocytic sarcoma, nodular lymphocyte predominant Hodgkin lymphoma, secondary histiocytic sarcoma, transdifferentiation

## Abstract

Histiocytic sarcoma is a very rare aggressive neoplasm of mature histiocytes which may present as a primary malignancy or transforming from a primary B-cell lymphoma that includes chronic lymphocytic leukemia/small lymphocytic lymphoma, follicular lymphoma, and extra nodal marginal zone lymphoma. A 69-year-old female presented with lymphadenopathy, and CT scan of chest, abdomen, and pelvis revealed extensive lymphadenopathy. Left axillary lymph node excision was performed. Histologic sections showed enlarged lymph nodes with architectural effacement by nodular and diffuse infiltrate comprising a mixture of small lymphocytes, histiocytes, occasional plasma cells, and scattered large atypical lymphocytes with irregular nuclear contours, vesicular chromatin, and prominent nucleoli. In addition, there were a few nodules of atypical histolytic cells including epithelioid and spindled forms and scattered large multinucleate forms. Immunohistochemical (IHC) stains showed that the large atypical B-cells were positive with variable intensity for CD20, PAX5, BCL6, BOB1 (weak), OCT2, MUM1, PU.1, CD45 (subset), CD19 (weak), CD79A (weak), and CD30 (subset, weak). They were negative for CD3, BCL2, CD15, ALK, CD10, IgD, HHV8, CAM5.2, EBER, GMS, AFB, SOX10, MART1, and HMB45. T-lymphocytes positive for CD3 showed rosette formation around scattered negative atypical large B-cells. CD21 and CD23 highlighted mild expansion of the follicular dendritic cell meshwork in a few areas of nodular infiltration by atypical cells. The nodules of atypical histiocytes were positive for CD68, CD163, BCL6, PU.1 (partial), cyclinD1, and S100 (partial) while negative for CD20, CD3, ALK, CD1A, HHV8, langerin, CAM5.2, HMB45, and MART1. The case was diagnosed as “Histiocytic sarcoma arising in a background of nodular lymphocyte predominant Hodgkin lymphoma.” Histiocytic sarcoma is a rare hematopoietic neoplasm, with limited cases of secondary histiocytic sarcoma transforming from a B-cell lymphoma reported in the English literature. Some of these case reports show the same clonal origin of histiocytic sarcoma and B-cell lymphoma. The diagnosis of the transformation is made based on the morphological, immunophenotypic, and genotypic features.

## 1. Introduction

Histiocytic sarcoma (HS) is a very rare, aggressive neoplasm of mature histiocytes that represents up to 1% of all neoplasm of the lymph nodes or soft tissues. It is seen in a broad age range, with a median age of 46 years, and there is a slight male predominance [1, 2]. Diagnosis is made by histopathology and confirmed by immunoreactivity for specific markers such as CD163, CD 8, and lysozyme [3–5]. HS may be seen as a primary malignancy or in association with another hematologic malignancy (secondary malignancy). Primary HS can affect solitary organs such as lymph nodes (less than 1%), skin, bone marrow, and spleen, as well as the nervous system and gastrointestinal tract [4, 6, 7]. Secondary HS has an aggressive course of disease compared to de novo HS [5]. Secondary HS is most commonly associated with a low-grade B-cell lymphoma; however, there are cases reported as secondary HS associated with B- and T-cell acute lymphoblastic leukemia/lymphoma [8]. There are several theories to explain the evolution of hematopoietic malignancies into HS, including (a) both of the tumors are originated from the same neoplastic progenitor, (b) mature lymphoid cell transdifferentiating to a myeloid lineage, and (c) mature lymphoid cell dedifferentiating to an immature progenitor cell, and subsequently redifferentiating to a myeloid lineage [2].

## 2. Case Report

A 69-year-old female presented to her primary care physician with complaints of discomfort in the left axillary region and was found with palpable lymph nodes on physical exam. The patient reported the presence of B symptoms, including fever, night sweats, and unintentional weight loss. Her medical history was notable for systemic lupus erythematosus. An ultrasound of the left axilla revealed multiple enlarged solid masses. Subsequent computed tomography (CT) scans of the chest and abdomen demonstrated extensive lymphadenopathy involving nodal regions both above and below the diaphragm. A positron emission tomography–computed tomography (PET-CT) scan revealed multiple fluorodeoxyglucose (FDG)-avid lymph nodes in the axillary, thoracic, abdominal, and retroperitoneal regions consistent with a high-grade lymphoma with widespread nodal involvement. No definitive evidence of extra nodal disease was identified on imaging. Left axillary lymph node excision was performed.

**Figure 1:**
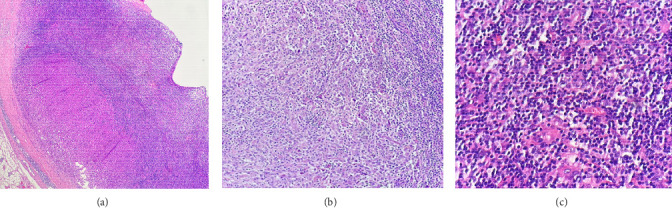
(a) Lymph node architectural effacement by a diffuse and nodular proliferation (H-E; 4x). (b) HS, atypical histiocytic cells include epithelioid and spindle forms (H-E; 40x). (c) B-cell lymphoma, diffuse infiltration (H-E; 40x).

Histologic sections showed enlarged lymph nodes with architectural effacement by a nodular and diffuse infiltrate (Figure 1(a)) comprising a mixture of small lymphocytes, histiocytes, occasional plasma cells, and scattered large atypical lymphocytes with irregular nuclear contours, vesicular chromatin, and prominent nucleoli (Figure 1(b)). In addition, there were a few nodules of atypical histiocytic cells including epithelioid and spindled forms with abundant eosinophilic cytoplasm and vesicular chromatin along with scattered large multinucleate forms (Figure 1(c)).

**Figure 2:**
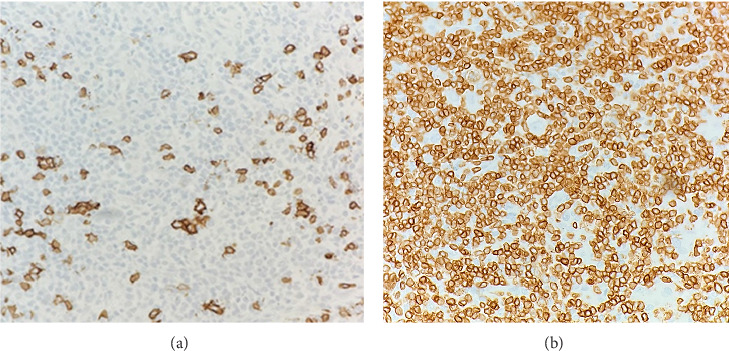
(a) B-cell lymphoma, large atypical cells positive with CD20 (10x). (b) B-cell lymphoma, large atypical cells are negative with CD3. Rosette formation of CD3 positive T-lymphocytes surrounding the atypical cells (20x).

**Figure 3:**
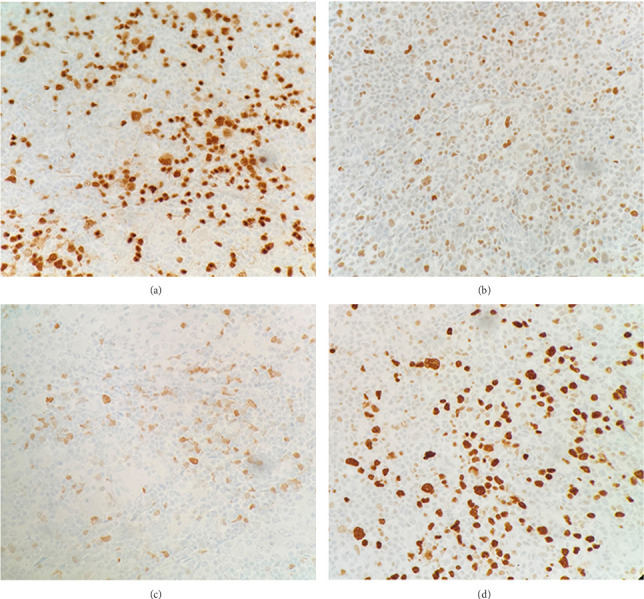
B-cell lymphoma, large atypical cells are positive for (a) PAX-5, (b) BCL-6, (c) BOB-1, and (d) OCT-2 at 10x.

**Figure 4:**
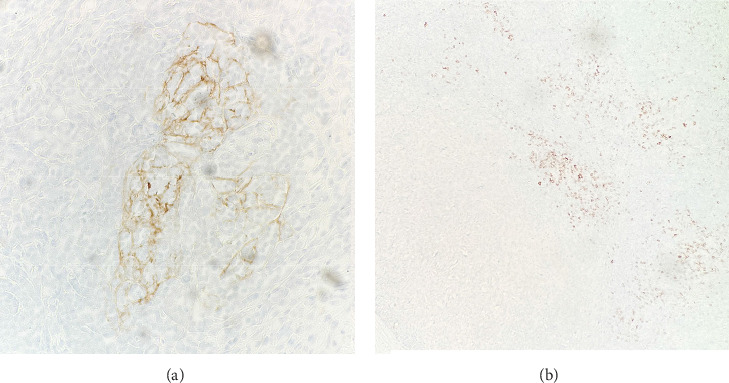
(a) Mild expansion of follicular dendritic cell meshwork (CD21; 20x). (b) Mild expansion of follicular dendritic cell meshwork, sparing the HS nodule on the bottom left (CD23; 4x).

**Figure 5:**
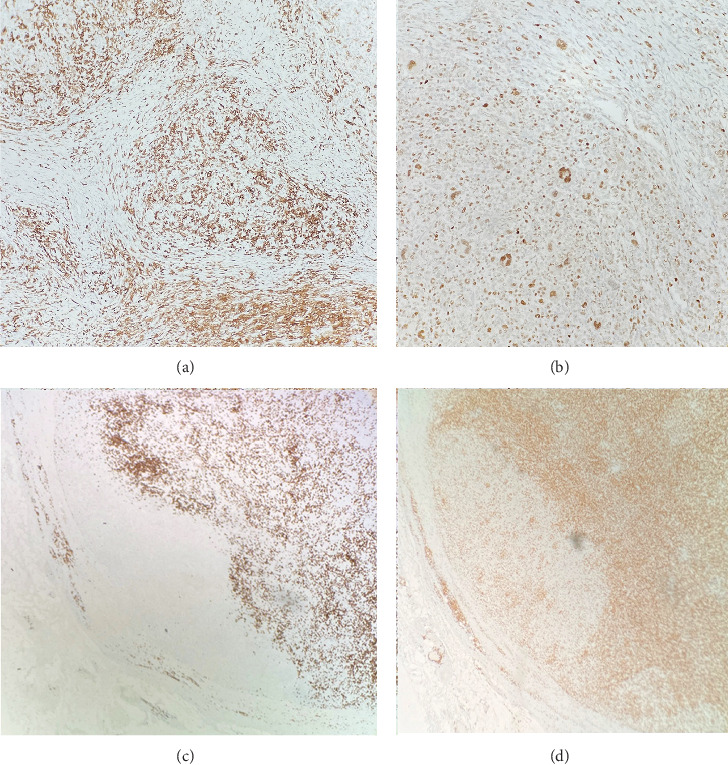
HS, atypical histiocytic cells are positive for (a) CD68 and (b) CD163 at 10x. HS, atypical histiocytic cells are negative for (c) CD20 and (d) CD3 at 10x.

**Figure 6:**
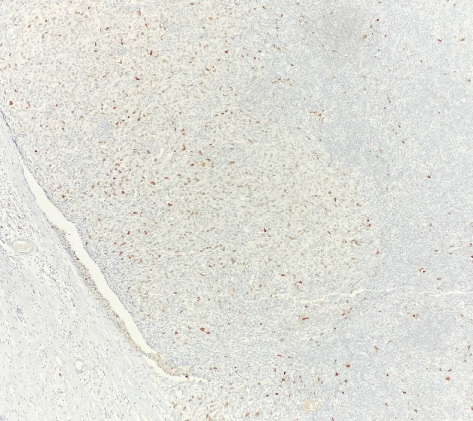
HS nodules show increased Cyclin D1 expression compared to the background adjacent NLPHL infiltrate at 10x.

Immunohistochemical (IHC) stains showed that the large atypical B-cells were positive with variable intensity for CD20 (Figure 2(a)), PAX5 (Figure 3(a)), BCL6 (Figure 3(b)), BOB1 (weak) (Figure 3(c)), OCT2 (Figure 3(d)), MUM1, PU.1, CD45 (subset), CD19 (weak), CD79A (weak), and CD30 (subset, weak). They were negative for CD3, BCL2, CD15, ALK, CD10, IgD, HHV8, CAM5.2, EBER, GMS, AFB, SOX10, MART1, and HMB45. T-lymphocytes positive for CD3 showed rosette formation around scattered negative atypical large B-cells (Figure 2(b)). Although the nodular architecture of nodular lymphocyte predominant Hodgkin lymphoma (NLPHL) is not readily apparent on the H-E stained sections, CD21 and CD23 highlighted mild expansion of the follicular dendritic cell meshwork in a few areas of nodular infiltration by atypical cells (Figure 4). The nodules of atypical histiocytes were positive for CD68 (Figure 5(a)), CD163 (Figure 5(b)), BCL6 (strong), PU.1 (partial), cyclinD1 (Figure 6), and S100 (partial) while negative for CD20 (Figure 5(c)), CD3 (Figure 5(d)), ALK, CD1A, HHV8, langerin, CAM5.2, HMB45, and Mart 1.

Considering the presence of scattered atypical large B-cells positive for BCL6, MUM1, BOB1, and OCT2 staining with rosette formation by CD3 positive cells accompanied by focal/scattered areas of preserved follicular dendritic cells, the findings are consistent with a NLPHL.

Flow cytometry showed no overt immunophenotypic evidence of non-Hodgkin B-cell lymphoproliferative disease. T-cells showed an increased CD 4/CD 8 ratio.

Fluorescence in situ hybridization (FISH) studies for MYC, BCL6, BCL2, Cyclin D1 (CCND1), MALT1, and BRAF were negative.

The morphologic and IHC findings identified a few nodules of markedly atypical histiocytes, consistent with HS. The surrounding lymph node was effaced by an atypical lymphoid infiltrate, consistent with a NLPHL.

At the time of diagnosis, the patient was clinically Stage IIIB, with Lymphocyte-Predominant International Prognostic Score (LP-IPS) of two [9]. She was treated with six cycles of R-CHOP (rituximab, cyclophosphamide, doxorubicin, vincristine, and prednisone) chemotherapy. A follow-up PET-CT after the completion of therapy demonstrated no evidence of metabolically active high-grade lymphoma, indicating a complete metabolic response. She achieved complete resolution of her B symptoms and remained asymptomatic with no evidence of disease at last follow-up 18 months after the initial diagnosis. She continues to be followed up regularly in the outpatient setting to monitor for disease recurrence or late treatment-related complications.

## 3. Discussion

HS is a very rare hematopoietic neoplasm, and there are limited cases of HS occurring in combination with B-cell lymphoma in the same lymph node. To our knowledge, there is not any HS in combination with NLPHL reported in the English literature [2, 8, 10–12].

There are six growth patterns of NLPHL depending on the histological and immunophenotypic features, Patterns A–F. The most common pattern is Pattern A, which is rich for typical B-cells. Most commonly, a mixture of growth patterns is seen. In Pattern E, a predominantly diffuse T-cell/histiocyte–rich large B-cell lymphoma (THRLBCL)–like pattern is seen. For these cases, at least one unequivocal NLPHL nodule is required to distinguish this variant pattern from de novo THRLBCL. Our case shows a predominant Pattern E with a minor component of Pattern A [7].

The most likely mechanism for this combination is mature B-cells transdifferentiating into myeloid lineage. It is believed that B-cells bypass the progenitor stage and transform into phenotypically distinct cells while retaining the same genotypic characteristics through genetic or epigenetic mechanisms. However, it is still not well understood [2].

Even though molecular features of HS are not very distinctive, secondary HS cases associated with B-cell lymphoma that exhibit transdifferentiation may share molecular aberrations with the B-cell lymphoma [4, 8].

In previous studies, secondary HS cases had clonal similarities with the related lymphoid neoplasia through IG or TRG gene rearrangements, or IGH/BCL2 sequence similarities. Most common mutations reported in the previous studies were RAS/MAPK pathway mutations, such as KRAS, BRAF, NF1, NRAS, and MAP21K1 [10, 11, 13, 14].

Also, it has shown that under the influence of transcription factors, such as C/EBPα, C/EBPβ and PU.1, B-cells can be programmed into macrophage/dendritic cells. Lack of PAX 5 expression also plays an important role in this process. In essence, overexpression of C/EBPα, C/EBPβ, or underexpression of PAX 5 can lead to transdifferentiation of B-cell tumor to a myeloid tumor, including HS [13, 14].

CCND1 protein is a major target of the MAPK signaling pathway. Recent studies have documented increased nuclear CCND1 expression disclosed by immunohistochemistry in histiocytic proliferations, correlating strongly with phosphorylated-ERK (p-ERK) expression and with MAPK pathway gene mutations [15]. In this case, HS nodules exhibit increased CCND1 immunostaining compared to the adjacent NLPHL infiltrate (Figure 6). This differential expression may suggest the presence of a mutation in the MAPK signaling pathway.

Since HS is a very rare disease, there is not a standard management protocol yet. The most common therapy for advanced disease is the CHOP protocol (cyclophosphamide, doxorubicin, vincristine, and prednisone), ICE protocol (ifosfamide, carboplatin, and etoposide), and bendamustine regimen. The discovery of specific driver mutations may lead to targeted therapies becoming the standard therapies for improving patient outcomes in the future [16].

Although FISH analysis for MYC, BCL6, BCL2, CCND1, MALT1, and BRAF were performed and yielded normal results, additional molecular studies comparing the two distinct tumors types were deemed not contributory by the treating oncologist. The lack of comprehensive molecular profiling of the HS and NLPHL components precludes a definitive assessment of the potential clonal relationship between the two.

The diagnosis of this transformation is made based on the morphology, immunophenotypic profile, and genetic features [11]. The findings of HS therefore necessitate an investigation to exclude an underlying associated malignancy.

## 4. Conclusion

We present the first secondary HS arising in a background of NLPHL in the English literature. Both HS and NLPHL are very rare entities, which exist in the same lymph node in our case.

## Data Availability

The data that support the findings of this study are available from the corresponding author upon reasonable request.
